# Placental microRNAs in pregnancies with early onset intrauterine growth restriction and preeclampsia: potential impact on gene expression and pathophysiology

**DOI:** 10.1186/s12920-019-0548-x

**Published:** 2019-06-27

**Authors:** Zain Awamleh, Gregory B. Gloor, Victor K. M. Han

**Affiliations:** 1 Children’s Health Research Institute, 800 Commissioners Road East, London, ON N6C 2V5 Canada; 2 Department of Biochemistry, The University of Western Ontario, London, ON N6A 3K7 Canada

**Keywords:** Intrauterine growth restriction, MicroRNA, Next-generation sequencing, Placenta, Preeclampsia

## Abstract

**Background:**

A normally developed placenta is integral to a successful pregnancy. Preeclampsia (PE) and intrauterine growth restriction (IUGR) are two common pregnancy related complications that maybe a result of abnormal placental development. Placental microRNAs (miRNAs) have been investigated as potential biomarkers for these complications, as they may play a role in placental development and pathophysiology by influencing gene expression. The purpose of this study is to utilize next-generation sequencing to determine miRNA and gene expression in human placental (chorionic villous) samples from three distinct patient groups with early-onset (EO) PE, IUGR, or PE + IUGR.

**Methods:**

Placental tissues were collected from four patient groups (control [*N* = 21], EO-PE [*N* = 20], EO-IUGR [*N* = 18], and EO-PE + IUGR [*N* = 20]), and total RNA was used for miRNA and RNA sequencing on the Illumina Hiseq2000 platform. For stringent differential expression analysis multiple analysis programs were used to analyze both expression datasets in each patient group compared to gestational age-matched controls.

**Results:**

Analysis revealed miRNAs and genes that are disease-specific, as well as others that were common between disease groups, which suggests common underlying placental pathologies in EO-PE and EO-IUGR. More specifically, 6 miRNAs and 22 genes were identified to be differentially expressed in all three patient groups. In addition, integrative analysis between the miRNA and gene expression datasets revealed candidate gene targets for miRNAs of interest.

**Conclusions:**

Integration of miRNA and RNA profiling in the same three subgroups of pregnancy complications, provides an alternate level of molecular information, in addition it can be used to better understand both unique and common molecular mechanisms involved in the pathophysiology of these diseases.

**Electronic supplementary material:**

The online version of this article (10.1186/s12920-019-0548-x) contains supplementary material, which is available to authorized users.

## Background

Abnormal placental development in pregnancy may result in complications such as preeclampsia (PE) and intrauterine growth restriction (IUGR) [1, 2]. Preeclampsia is a maternal pregnancy disorder characterized by hypertension and proteinuria, and occurs in 2–8% of pregnancies worldwide [3, 4]. Intrauterine growth restriction is poor fetal growth in utero with an expected fetal weight lower than the 10th percentile estimated for gestational age and gender, associated with abnormal Dopplers in fetal and umbilical vessels [5, 6].

PE and IUGR are heterogeneous in etiology and can be attributed to maternal, fetal and/or placental factors. Maternal risk factors associated with PE include maternal diabetes, pre-existing hypertension and obesity [4]. These maternal risk factors have also been found to be associated with IUGR [5]. IUGR is also associated with fetal risk factors such as chromosomal abnormalities, congenital anomalies and infection [5, 6]. A subset of patients develop early-onset PE together with IUGR, suggesting an overlap in the etiology underlying these complications. Histopathology of placentae from PE and IUGR pregnancies shows similar microscopic placental abnormalities, supporting the concept of similar pathophysiology underlying these two disorders [7].

Although the gestational age of the onset of signs and symptoms is based on clinical outcomes and there is some overlap, it is now recognized that the early- and late- onset forms of the diseases may have different pathophysiology [8]. Early-onset forms of these complications are more severe, where the mother and infant are at higher risk of short- and long-term adverse health outcomes [6, 9]. In PE pregnancies, mothers experience a systemic disturbance mainly characterized by hypertension and proteinuria, and are at higher risk of cardiovascular disease later in life [3, 4]. Meanwhile, the newborns are usually born prematurely and are impacted by the mortality and morbidity of preterm birth in both PE and IUGR pregnancies.

Placental micro (mi)RNAs have been investigated for their role in the growth and function of the placenta, and for their potential use as diagnostic biomarkers due to their ability to enter maternal circulation and are detectable in the maternal plasma [10]. Epigenetics is defined as the mechanisms regulating gene expression without changes to the DNA sequence. Research has identified a number of epigenetic mechanisms including DNA methylation, histone modifications and non-coding RNAs. MiRNAs are a class of non-coding RNAs that can be tissue-specific and are encoded in the human genome [11, 12]. The regulation of gene expression by miRNAs by targeting mRNAs to transiently block translation or degrade the mRNA without altering the DNA sequence, classifies miRNAs as epigenetic regulators [11, 12]. The final product of miRNA biogenesis is an 18–22 base pair, single nucleotide strand, transcribed in the nucleus and then transported into the cytoplasm [11, 12]. Placenta-specific clusters have been identified on chromosomes 14 and 19 [13, 14]. Differential expression analysis of miRNAs in the placenta has revealed a dysregulation in miRNA expression in placentae from pregnancies complicated with PE and IUGR [15, 16]. The most consistent finding is the increased expression of miR-210 in PE placentae [15]. However, there are differences in findings among previous studies that can be attributed to: (i) the definition of patient groups; (ii) differences in the platforms used to measure expression; and (iii) analytical methods used to identify differentially expressed miRNAs.

The purpose of this study is to utilize next-generation sequencing (NGS) to identify miRNAs and genes expressed in the same placental tissues from three patient groups with early-onset diseases. The patient groups include: PE only, IUGR only, PE + IUGR, and gestational age-matched controls without PE or IUGR. This analysis aims to identify differentially expressed miRNAs and genes in each disease group compared to the control group, and to integrate the two expression datasets to identify potential gene targets regulated by miRNAs.

## Methods

### Sample collection

Preeclampsia was defined as hypertension (blood pressure > 140/90 mmHg) and proteinuria (≥ 300 mg in 24 h) [3, 4]. Intrauterine growth restriction was defined as estimated fetal weight below the 10th percentile for gestational age and gender, associated with abnormal umbilical and uterine artery Doppler evaluations [5, 6]. Patients with PE + IUGR presented with criteria aforementioned for both diseases. Only patients diagnosed prior to 34 weeks (early-onset) were included in this study. Patients with preterm labor and no other complications before 34 weeks of gestation were recruited as controls. Women with diabetes, gestational diabetes, pre-existing hypertension, obvious chorioamnionitis, alcohol/drug use, chromosomal or genetic abnormalities, congenital anomalies, or infection were excluded. All women enrolled in this study gave written informed consent for the collection of samples and information. This research was approved by the office of Human Research Ethics at Western University (REB # 102621). Samples were collected from two central and two peripheral portions of the placenta within 30 min of delivery. Central samples were collected within a 5 cm radius from the umbilical cord insertion site and the peripheral samples were collected 2–3 cm from the edge of the placenta. Full depth 1 cm × 1 cm tissue samples were excised, and the maternal decidua was separated from the chorionic villi using gross dissection. In this study, the maternal and fetal components were separated and only the fetal components (chorionic villi) were used for analysis. The tissue samples were flash frozen in liquid nitrogen and stored in − 80 °C until further analysis.

### RNA isolation and sequencing

Total RNA was isolated from 80 to 100 mg of tissue samples from each of the four regions of each placenta using the mirVana RNA isolation kit (Life Technologies, Waltham, MA, USA). Sample quantity and quality was checked using the Agilent Bioanalyzer 2100 (Agilent Technologies, Palo Alto, CA, USA). Total RNA isolated from central and peripheral samples of each placenta was pooled in equal quantities for one representative total RNA sample from each patient. Samples were then sent to the Génome Québec Innovation Centre at McGill University (Montreal, QC, Canada) for library preparation and sequencing. The Illumina Truseq RNA and smRNA library preparation kits were used to prepare mRNA and miRNA libraries respectively (Illumina, San Diego, CA, USA). Samples were sequenced on the Illumina HiSeq 2000.

### Differential expression analysis

Using R v3.3.1 [17], three Bioconductor packages were used for differential expression analysis, DESeq2 [18] (Benjamini-Hochberg adjusted *p*-value < 0.01), edgeR [19] (Benjamini-Hochberg adjusted *p*-value < 0.01) and ALDEx2 [20] (effect > 0.8). Samples that contributed at least the median plus twice the inter-quantile range (IQR) of variance to the group were considered outliers and were subsequently removed from the analysis [21]. Only miRNAs and genes identified by at least two programs were considered significant. To adjust for covariates (fetal sex, maternal BMI, gestational age, and mode of delivery (labor/no labor, C-section/vaginal)), surrogate variable analysis (SVA R version 3.6) [22] was used with DESeq2 and edgeR. The miRNA and gene expression data are available from the Gene Expression Omnibus (GEO) database under accession numbers GSE114349 and GSE114691, respectively.

### Validation of miRNAs using qRT-PCR

Total RNA was reverse transcribed using TaqMan advanced miRNA cDNA synthesis kit (Life Technologies, Waltham, MA, USA). Quantification of miRNAs was completed using the TaqMan fast advanced PCR master mix in conjunction with TaqMan miRNA expression assays (Life Technologies, Waltham, MA, USA). For miRNA normalization, miR-191-5p was used as an endogenous control [23, 24]. The ΔΔC_t_ method was used for analysis of real-time data to obtain a miRNA fold-change relative to miR-191-5p expression. Mann-Whitney-U was used for statistical analysis.

### Target prediction and gene ontology

Spearman correlation co-efficient was used for correlation analysis between miRNA and gene expression values. Only correlation with a r_s_ ≤ − 0.5 and *p*-value ≤0.01 was considered significantly negatively correlated. To further refine results only genes with a fold-change ≤ − 1.5 or ≥ 1.5 were included for target prediction analysis. Two target prediction software were used: TargetScan (v7.1) [25] and DIANA-microT-CDS (v5.0) [26]. Criteria used for final selection of miRNA gene targets is in Additional file 1: Table S1. Gene ontology analysis was completed using WebGestalt (2013) [27].

## Results

### Patient selection criteria and principal component analyses

Our aim was to select patients with primarily placental factors underlying the diseases, therefore stringent inclusion and exclusion criteria were used in patient recruitment into the study. Patients with known maternal and/or fetal risk factors were not included (see methods). Clinical characteristics of the patient cohorts are described in Table 1. There were no differences in maternal age, BMI or gestational age at delivery between patient groups. There were significant differences in birth, placental weights and blood pressure between patient groups with complicated pregnancies and gestational age-matched controls.

**Table 1:**
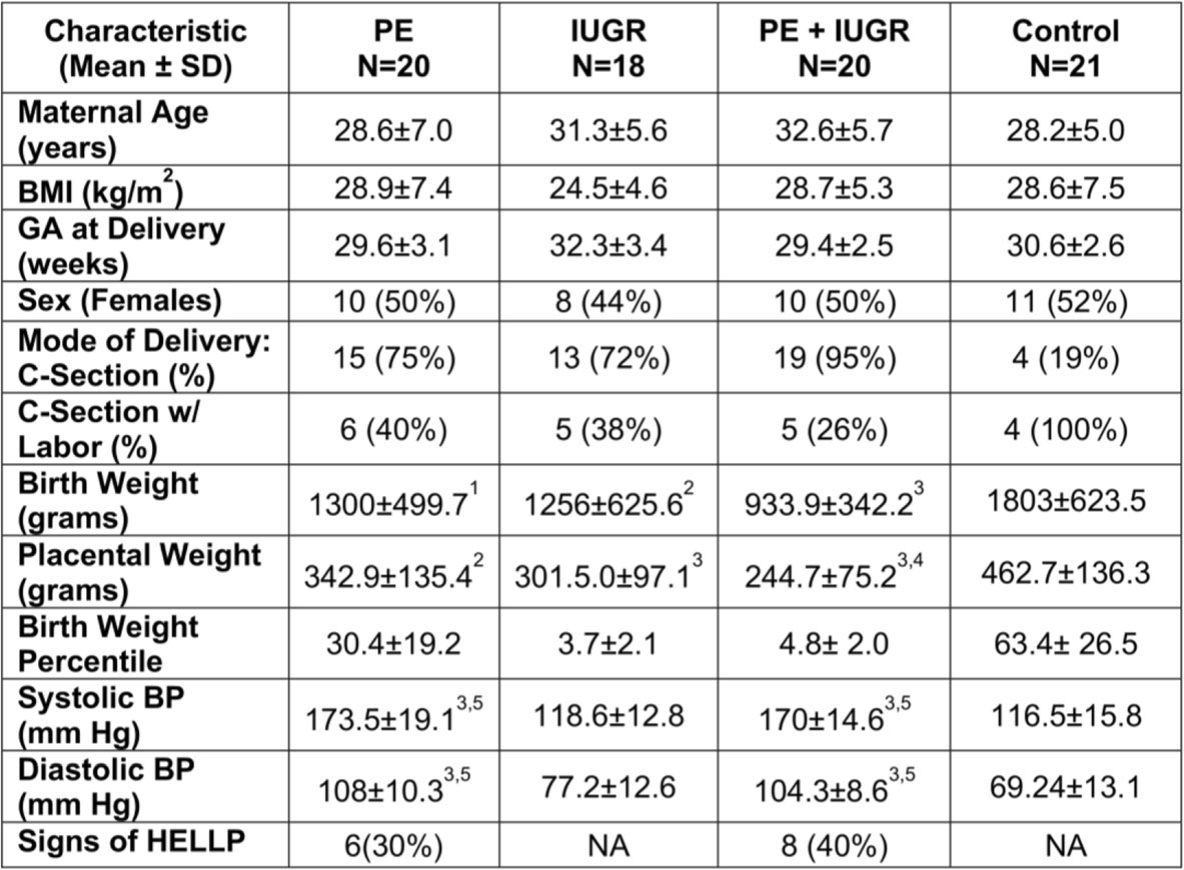
Clinical characteristics of the patient groups with complicated pregnancies and gestational age matched controls

1) *p*-value < 0.05 vs. control 2) *p*-value < 0.001 vs. control 3) *p*-value < 0.0001 vs. control 4) *p*-value < 0.01 vs. PE only 5) *p*-value < 0.0001 vs. IUGR only

Preliminary exploratory analysis of the miRNA and gene expression datasets had two aims: identification of outliers and principal component analysis (PCA) to determine if the samples separated by disease. Identified outliers were removed prior to PCA. Based on miRNA expression data sets, one patient each was removed from the PE only group (*N* = 19) and from the control group (*N* = 20). Two patients were removed from the IUGR group (*N* = 16), and none were removed from the PE + IUGR group (*N* = 20). Based on the gene expression data set, one patient was removed from the PE only group (*N* = 19), three patients from the IUGR only group (*N* = 15), and four patients from the PE + IUGR group (*N* = 16). None were removed from the control group (*N* = 21).

Principal component analysis was then used to determine how patient samples cluster based on the expression data. Based on expression of all known human miRNAs, samples clustered separately based on disease status on the first principal component (Additional file 2: Figure S1 a-c). Based on the transcriptome, samples also clustered separately based on disease status on the first principal component (Additional file 2: Figure S1 d-f). Patients with PE and IUGR clustered away from controls with 15–40% of the variance in the data explained by the first principal component.

### Differential expression analysis of placental miRNAs

**Fig. 1:**
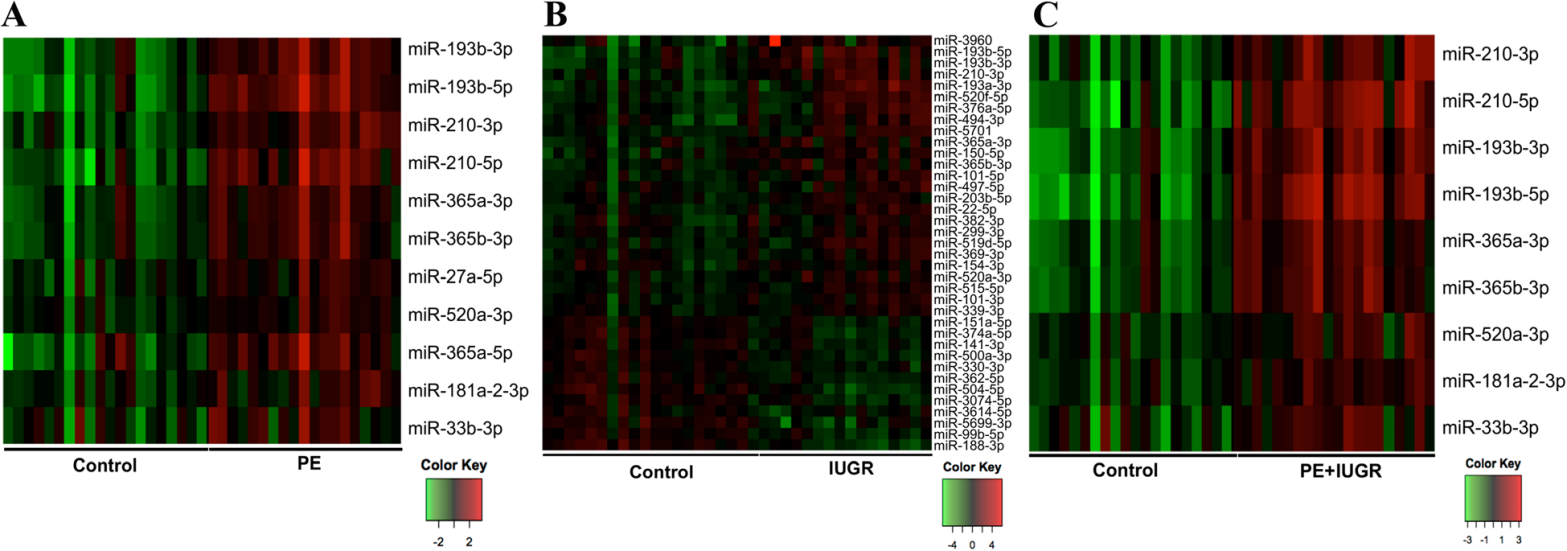
Heat maps depicting differentially expressed microRNAs in each patient group. Differential expression analysis revealed 11 upregulated miRNA(s) in (**a**)**.** Preeclamptic placenta 25 upregulated and 12 downregulated miRNAs in (**b**)**.** Intrauterine growth restricted samples, and 9 upregulated miRNAs in (**c**)**.** Preeclamptic and Intrauterine growth restricted samples. Three programs were used for differential expression analysis, and only miRNAs identified by two or more programs were included in the final results

**Fig. 2:**
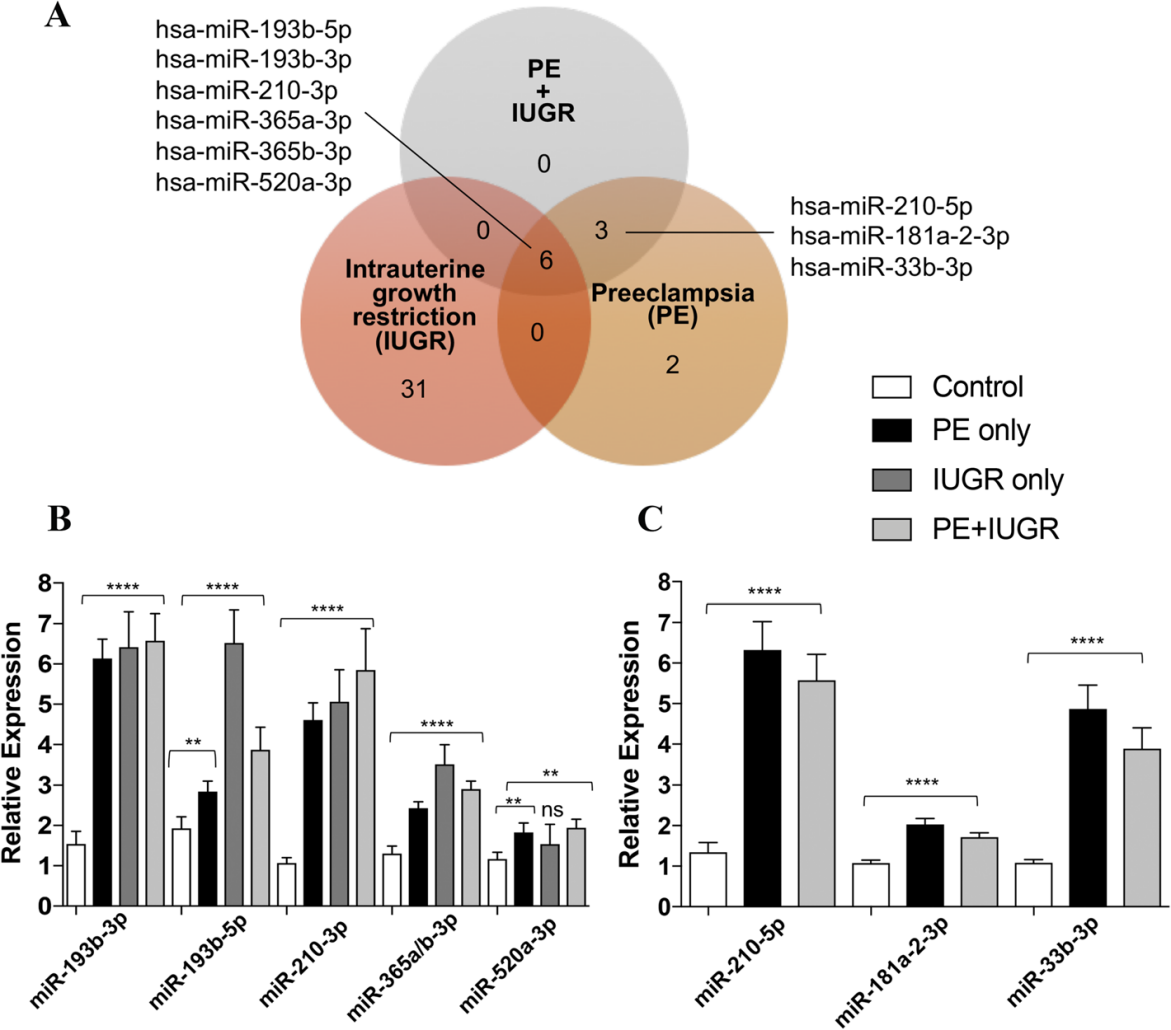
Comparing differentially expressed miRNAs between patient groups. **a** Six miRNAs are found to be common to all three patient groups, whereas three miRNAs are specific to patients with preeclampsia. **b** Validation of miRNAs common to all three patient groups (with the exception of miR-520a-3p in the IUGR only group), and **c** miRNAs common to preeclamptic patients using qRT-PCR. To find relative expression, delta-delta C_T_ was used and values were normalized to hsa-miR-191-5p expression. Data is shown as the mean ± S.E.M, significant differences were determined by a Mann-Whitney U test. ****p-val < 0.0001 **p-val < 0.01

To identify differentially expressed miRNAs in each patient group compared to gestational age- matched controls, three different programs namely DESeq2, edgeR, and ALDEx2 were used. Only miRNAs identified by at least two were considered significant. Using DESeq2 and edgeR, we accounted for covariates in the model such as fetal sex, maternal BMI, gestational age, and mode of delivery. We identified 11 up-regulated miRNAs in the PE only samples (Fig. 1a), 25 upregulated and 12 downregulated miRNAs in IUGR samples (Fig. 1b), and 9 upregulated miRNAs in PE + IUGR samples (Fig. 1c) (Additional file 3: Table S2). Comparison of all differentially expressed miRNAs, revealed 6 miRNAs that are common to all three patient groups (Fig. 2a). All of the differentially expressed miRNAs were confirmed using qRT-PCR, with the exception of miR-520a-3p in the IUGR group (Fig. 2b). Similarly, 3 miRNAs that were common to patients with PE only (Fig. 2a) were also validated using qRT-PCR (Fig. 2c).

### Differential gene expression analysis in the patient cohorts

**Fig. 3:**
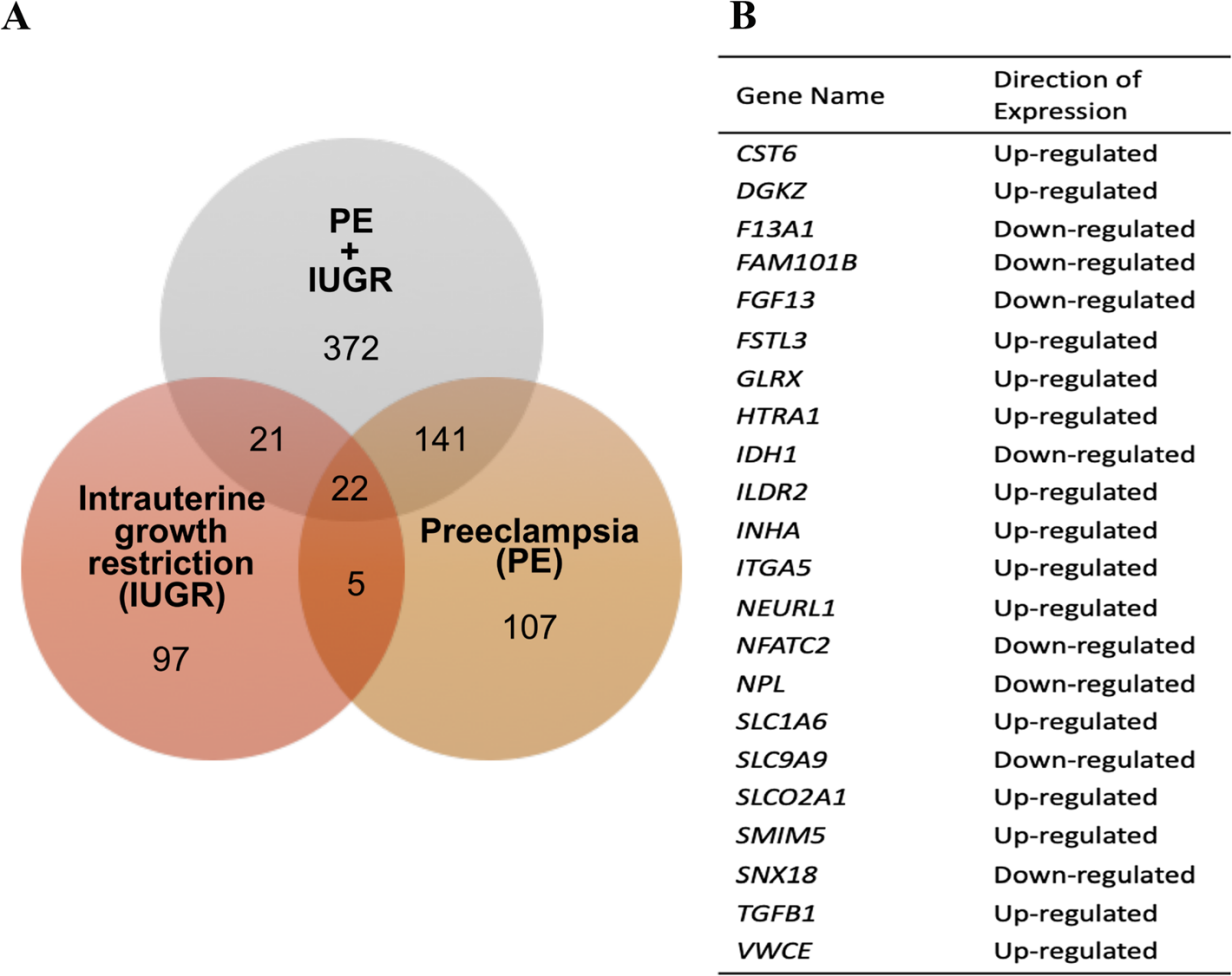
Comparing differentially expressed genes between patient groups. **a** Venn diagram shows the number of differentially expressed genes in each patient group compared to the control group. Three programs were used for differential expression analysis (DESeq2, edgeR, ALDEx2), and only genes identified by two or more programs were considered differentially expressed. **b** This analysis revealed 22 genes common to all three patient groups

The same differential expression analysis methods used for the miRNA dataset were applied to the gene expression dataset. In total, there were 275 differentially expressed genes in the PE only samples, 155 differentially expressed genes in the IUGR only samples, and 556 differentially expressed genes in PE + IUGR samples (Additional file 4: Table S3). Top ten up- and down- regulated genes in each disease group are shown in Additional file 5: Figure S2 (a-c). Comparison of differentially expressed genes in all patient groups revealed 22 differentially expressed genes in placental samples from all three disease groups (Fig. 3). Other genes showed specificity to patients with PE or patients with IUGR, with 141 and 21 differentially expressed respectively. Lists of common genes between patient groups are found in Additional file 6: Table S4.

### Target prediction and gene ontology analyses

**Fig. 4:**
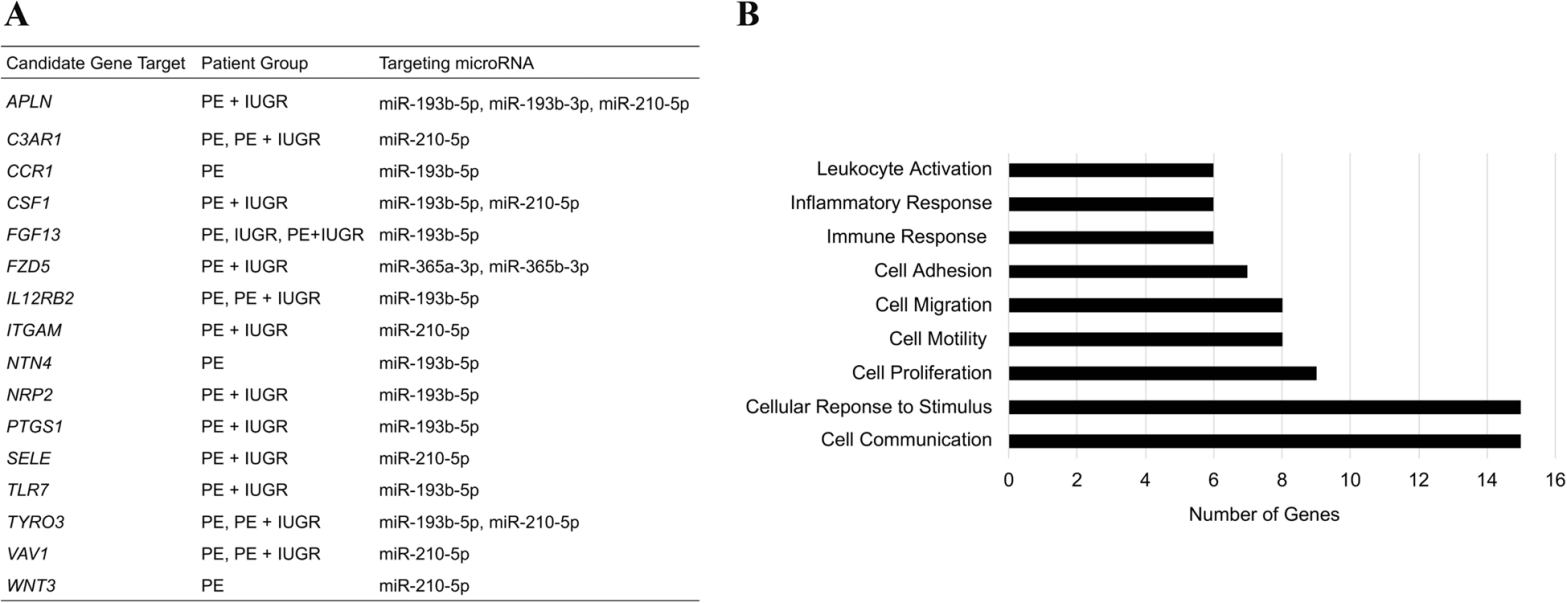
Target prediction and gene ontology analyses for all patient groups. **a** Identified candidate gene targets for miRNAs that have been validated with qRT-PCR. Spearman correlation was used to identify genes that inversely correlate in expression to validated miRNAs, in each patient group. Candidate gene targets were identified using prediction software DIANA-microT-CDS and TargetScan. **b** Gene ontology analysis for identified candidate genes

As epigenetic regulators, abundantly expressed miRNAs can impact gene expression by targeting mRNAs in the cytoplasm. Inverse correlation analysis using Spearman’s correlation co-efficient identified genes that negatively correlated in expression to differentially expressed miRNAs. Identified genes had a significant negative correlation with one or more miRNA. To further refine the results, only candidate genes with fold-change (≤ − 1.5 or ≥ 1.5) were selected for further analysis. Target prediction software programs (DIANA-microT-CDS and TargetScan) were then used to identify predicted gene targets for miRNAs. A summary of criteria used to select appropriate gene targets is found in Additional file 1: Table S1. This analysis resulted in 16 candidate gene targets (Fig. 4a) (Additional file 7: Table S5). The majority of candidate gene targets identified were found in PE only or PE + IUGR groups. Interestingly, there were candidate genes such as *APLN*, *CSF1*, *FZD5*, and *TYRO3* that are potential targets of multiple miRNAs (Fig. 4a). Gene ontology (GO) analysis of the 16 candidate gene targets revealed that most genes are involved in important cellular functions including proliferation, migration, communication and adhesion (Fig. 4b).

## Discussion

Our study identified dysregulated miRNAs and genes in placental samples that are common to three patient groups with early-onset pregnancy complications. More specifically, 6 miRNAs and 22 genes were found to be commonly differentially expressed in patients with early-onset PE, IUGR, and PE + IUGR, compared to gestational age-matched controls. However, some miRNAs and genes remained disease specific. To integrate the miRNA and gene expression datasets, inverse correlation analysis revealed candidate gene targets primarily involved in cell functions such as proliferation and migration.

MicroRNAs identified to be common to all patient groups, are some of the most commonly identified miRNAs in the literature. MicroRNAs identified in this study have been identified in studies measuring miRNA expression in placental samples from primarily PE pregnancies. Studies assessing global miRNA expression in placenta from IUGR pregnancies are scarce, potentially due to heterogeneity of etiologies underlying this condition. Studies utilizing global miRNA expression analysis in PE with overlapping findings to this present study are found in Additional file 8: Table S6. One of the most well characterized miRNAs in pregnancy complications is miR-210 [15, 28]. Under hypoxic conditions, miR-210 is upregulated by the transcription factor HIF-1α [28]. The intrauterine environment in pregnancies complicated by PE and/or IUGR has been suggested to be hypoxic due to decreased perfusion of maternal blood to the feto-placental unit. In our study, we identified that miR-210-3p is common to all patient groups, whereas miR-210-5p is common to patients with PE (Fig. 2). Gene targets of miR-210 have also been investigated to identify the impact of the upregulation of miR-210 on the placenta during disease [28]. Identified targets for miR-210 include *EFNA3* and *HOXA9*, which are important for cell functions such as migration and invasion [28, 29]. In our study, downregulated predicted targets of miR-210-5p are also involved in cell migration, they include *APLN*, *CSF1*, *ITGAM*, *C3AR1*, and *SELE* (Fig. 4, Additional file 7: Table S5). These novel gene targets for miR-210-5p have been identified to be downregulated in patients with preeclampsia (± IUGR) in our study (Additional file 4: Table S3). Gene targets such as *APLN* and *C3AR1* have been shown in the literature to be down regulated in placental samples from PE patients with an unknown underlying mechanism for dysregulation [30, 31]. Apelin (*APLN*) is expressed in placental syncytiotrophoblast cells, and evidence shows the involvement of *APLN* in angiogenesis [30].

Other miRNAs such as miR-193b-3p/5p, and miR-365a/b-3p have also been previously identified in studies measuring miRNA expression in placental samples from PE pregnancies [32–34] (Additional file 8: Table S6). Identified gene targets for miR-193b-3p/5p are enriched in gene ontology categories important for cell functions such as cell proliferation, adhesion, and migration (Fig. 4a). Zhou et al. also showed increased expression of miR-193b-3p and -5p in PE patients, and that miR-193b-3p was found to decrease migration and invasion of HTR-8/SVneo cells [34]. We also identified *FZD5* (frizzled 5) as a candidate gene target for miR-365a/b-3p, which belongs to the frizzled family of proteins, known to be important during placental development (Fig. 4). Frizzled 5 mRNA and protein have also been reported to be down regulated in PE placenta [35]. In the IUGR only group the majority of miRNAs remained unique to that patient group (31/37). Of the 37 differentially expressed miRNAs in the IUGR only group, 4 belonged to the C19MC (520a-3p, 520f-5p, 515-5p, 519-5p) and 6 belonged to the C14MC (299-3p, 494-3p, 376a-5p, 382-3p, 154-3p, 369-3p), the two miRNA clusters known to be specific to the placenta [1]. Enrichment of gene targets in GO categories such as cell migration and proliferation is not a surprising finding since recent literature evidence has shown the role of miRNAs in these functions, in addition to cell invasion and differentiation [29, 34–36]. Findings are primarily reported in placental or cancer studies, since there are many parallels between placental and tumor development. In the placenta these are important trophoblast cell functions, impairment of these functions in trophoblast cells can result in placental maldevelopment and insufficiency. For example, intermediate cytotrophoblast cells must migrate and differentiate into extravillous trophoblast cells to then invade and remodel the maternal spiral arteries to establish maternal blood flow. This is a complex process and when impaired is thought to contribute to the etiology of PE and IUGR.

Comparison between patient groups revealed a subset of genes common to the three diseased patient groups. A number of the differentially expressed genes the we have identified in our diseased patient cohorts, have previously been identified to be dysregulated in placental samples from complicated pregnancies. One example includes *INHA* (inhibin) and *FSTL3* (follistatin-like 3), both of which have been previously reported to be upregulated in PE placenta [37, 38]. In this study *INHA* and *FSTL3* are upregulated in pregnancies with IUGR as well (Fig. 3, Additional file 4: Table S3). *FSTL3* expression is enhanced by hypoxia and plays a role in trophoblast differentiation, migration and invasion [39, 40]. *INHA* also plays a role in trophoblast differentiation, and syncytial fusion [41–43].

Differences observed between this current study and other studies assessing global expression can be attributed to (i) strict patient selection criteria, (ii) platform used to measure miRNA and gene expression, and (iii) methods used for differential expression analysis. Patients in this study were strictly of early-onset disease, and by excluding a number of maternal and fetal predisposing factors, the patients were better standardized as to the pregnancy complications [8]. Despite this current study being the largest to integrate miRNA and gene expression data in these patient cohorts to our knowledge, a larger sample number or additional replication groups would be beneficial. However, strict patient selection criteria were beneficial, this is demonstrated in principal component analyses based on miRNA and gene expression data in these patient groups that showed clear segregation by disease status on the first principal component. In addition, previous studies on PE do not always stratify patients into PE only and PE + IUGR, whereas in our study, these two patient populations were segregated and treated as distinct populations. Recent studies have shown distinct gene expression profiles for different subclasses of PE (late vs. early, ± IUGR) [44, 45]. Leavey at al. combined microarray gene expression data from previously published studies in PE placentae, and conducted unsupervised clustering analysis [45]. The study identified three main subclasses of PE based on gene expression - late-onset PE, which is mostly associated with term deliveries and maternal risk factors (“maternal PE”); early-onset PE, which is likely placental in origin and is more frequently associated with IUGR (“canonical PE”); and a third subclass of PE that is also severe and can co-occur with IUGR but is likely due to poor maternal-fetal compatibility (“immunologic PE”) [45]. Interestingly, cluster analysis based on DNA methylation data in PE placentae revealed a similar clustering pattern into the three subclasses of PE [46]. In this study, 8763 and 340 differentially methylated sites were found in the “canonical” and “immunologic” subclasses respectively [46]. Wilson et al. also showed differentially methylated sites, as many as 599 sites, in EO-PE and only 5 in late-onset PE [47]. These studies show differences in gene expression and in epigenetic mechanisms between subclasses of PE, emphasizing that each subclass has a unique underlying pathophysiology. In addition, these studies demonstrate the benefits of combining epigenetic and gene expression data to improve our understanding of molecular mechanisms in the placenta.

Next-generation sequencing (NGS) used in this study to assess miRNA and gene expression has many advantages compared to microarray platforms [48, 49]. The detection capacity of microarrays is limited by the pre-determined probes on the array platforms, whereas NGS data can be used to align to the most updated sequence information, which is beneficial for the discovery of new miRNAs and genes. Moreover, NGS has a wider dynamic range and is therefore capable of detecting miRNAs and mRNAs that are expressed at low levels. Interestingly, from our miRNA study, we identified that our findings overlap more with studies that have also used sequencing techniques compared to studies utilizing microarray technology [34, 50].

Lastly, the stringency of statistical analyses to identify differentially expressed miRNAs and genes is beneficial to increase confidence but could result in the exclusion of some miRNAs and genes that may contribute to pathogenesis of PE and/or IUGR. On the other hand, methods for analyzing high-throughput data are currently not standardized, particularly normalization and identification of differences [51]. Each program in this study utilized a different approach to the normalization and differential expression analysis. Therefore, using stringent cut-off values and including only miRNAs and genes identified by two or more programs increased the reliability of our findings compared to using a single approach for differential expression. Finally, the use of preterm labor patients as controls is a limitation to this study. Differential expression analysis in this study is conducted in comparison to gestational age-matched controls. Although it is beneficial to compare placental samples of similar gestational age because of the prime consideration of comparing placental tissues that are of similar developmental stages, controls in this study are from preterm births that may not be considered normal nor healthy since evidence shows that preterm births are a result of perturbations during gestation some of which can be attributed to infection and inflammation in the placenta. In the future, inclusion of both preterm and term controls may be beneficial for understanding how selection of control samples impacts results.

## Conclusion

In summary, this study shows that maldevelopment of the placenta early in gestation may manifest itself into different complex diseases in the second and third trimester, however some common underlying pathophysiological mechanisms remain in the placenta. Identification of common miRNAs and genes that are dysregulated in all three patient groups supports this observation. The gene expression data set allowed us to identify potential novel gene targets that are primarily involved in cellular processes important for placental growth and function. Identified candidate gene targets can be further experimentally validated to demonstrate miRNA-mRNA interactions and to assess the impact of miRNAs on gene expression. Since miRNAs can enter maternal circulation during pregnancy, there are studies assessing the diagnostic value of miRNAs in these pregnancy complications. However, due to heterogeneity of samples and differences between studies, no miRNAs with good diagnostic value have been identified. In this particular study, due to exclusion of many patients with maternal and fetal risk factors, miRNAs identified in this study may be helpful in identifying an obstetrical population at high risk of developing PE or IUGR, that do not have obvious maternal and fetal risk factors. Since the pathological processes precede the clinical signs and symptoms of PE or IUGR, it is possible that the miRNAs identified in this study may be altered in the maternal circulation early in pregnancy and may serve as potential biomarkers that may predict PE or IUGR. Future studies may include analysis of maternal plasma samples completed retrospectively by measuring miRNA levels in plasma samples obtained early in pregnancy as a part of routine clinical care to more accurately assess diagnostic value across gestation. Continued integration of epigenetic and gene expression data in larger disease cohorts can validate findings and expand our understating of molecular mechanisms impacting placental growth and function.

## Additional files

Additional file 1: **Table S1.** Criteria for selection of appropriate gene targets for microRNAs. (TIF 2 kb)

Additional file 2: **Figure S1.** Principal Component analysis (PCA) plots based on miRNA and gene expression datasets. (TIF 17 kb)

Additional file 3: **Table S2.** List of differentially expressed microRNAs in each disease group compared to gestational age-matched controls including significance values from each program and direction of expression. (XLSX 11 kb)

Additional file 4: **Table S3.** List of differentially expressed genes in each disease group compared to gestational age-matched controls including significance values from each program and direction of expression. (XLSX 55 kb)

Additional file 5: **Figure S2.** Heat maps of top differentially expressed genes in each patient group. (TIF 12 kb)

Additional file 6: **Table S4.** List of genes common between: PE Only and PE + IUGR, PE Only and IUGR Only and PE + IUGR and IUGR Only. (XLSX 12 kb)

Additional file 7: **Table S5.** Identified candidate gene targets based on inverse correlation analysis including correlation coefficients and gene fold changes. (XLSX 11 kb)

Additional file 8: **Table S6.** List of global microRNA expression studies in human placenta from preeclamptic patients compared to this current study. (TIF 1 kb)

## Data Availability

MicroRNA and gene expression data for all patients generated in this study are available through the Gene Expression Omnibus (GEO) database under accession numbers GSE114349 and GSE114691, respectively.

## Abbreviations

EO: Early-onset
IUGR: Intrauterine Growth Restriction
NGS: Next-Generation Sequencing
PCA: Principal Component Analysis
PE: Preeclampsia
qRT-PCR: Quantitative Real time PCR
