# Supplementary figures and images for: Placental microRNAs in pregnancies with early onset intrauterine growth restriction and preeclampsia: potential impact on gene expression and pathophysiology
Source: BMC Med Genomics. 2019 Jun 27;12:91. doi: 10.1186/s12920-019-0548-x (PMC6598374; doi:10.1186/s12920-019-0548-x)

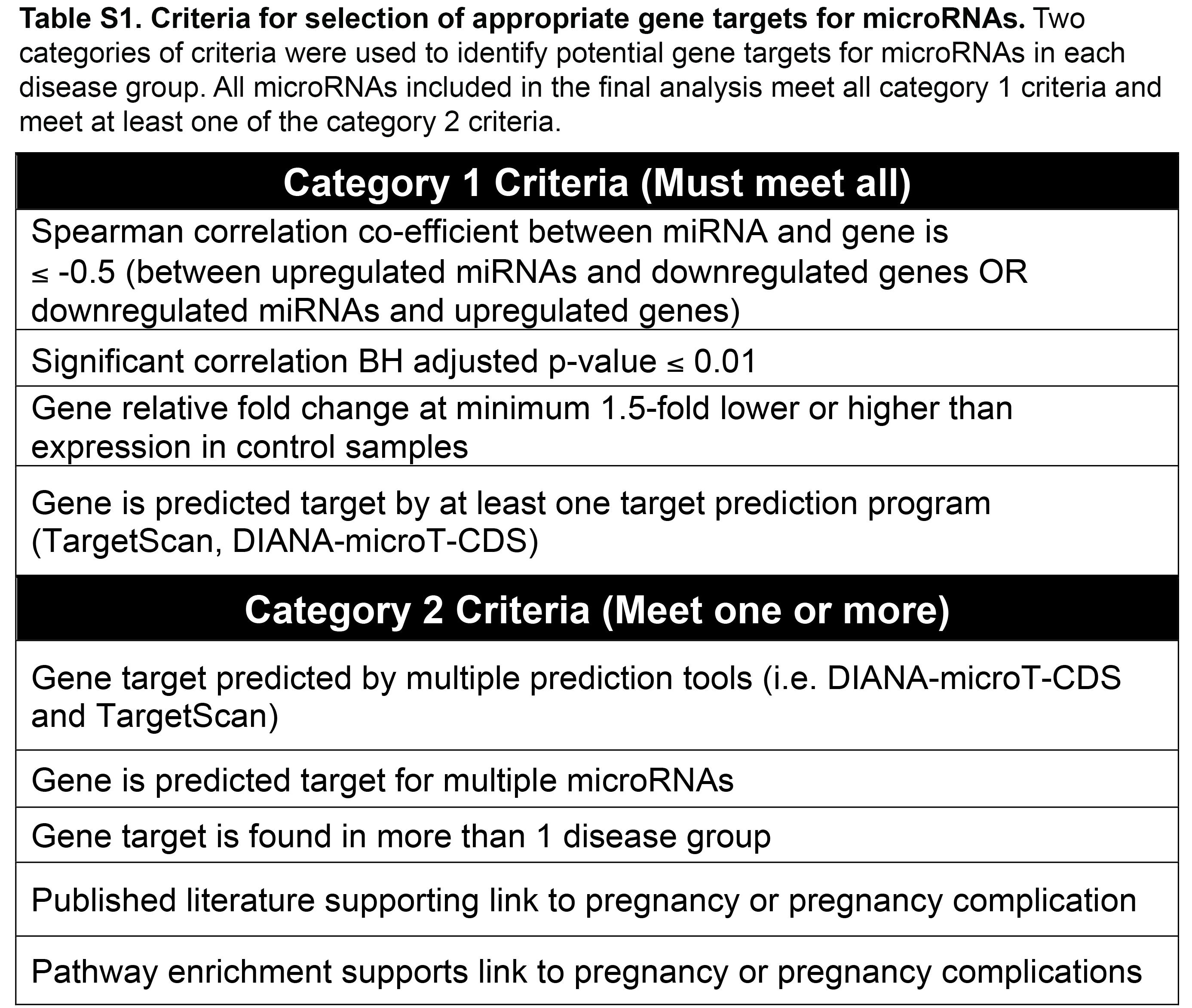

Supplement: Supplementary file 1 — Table S1. Criteria for selection of appropriate gene targets for microRNAs. (TIF 2 kb) [file 12920_2019_548_MOESM1_ESM.tif]

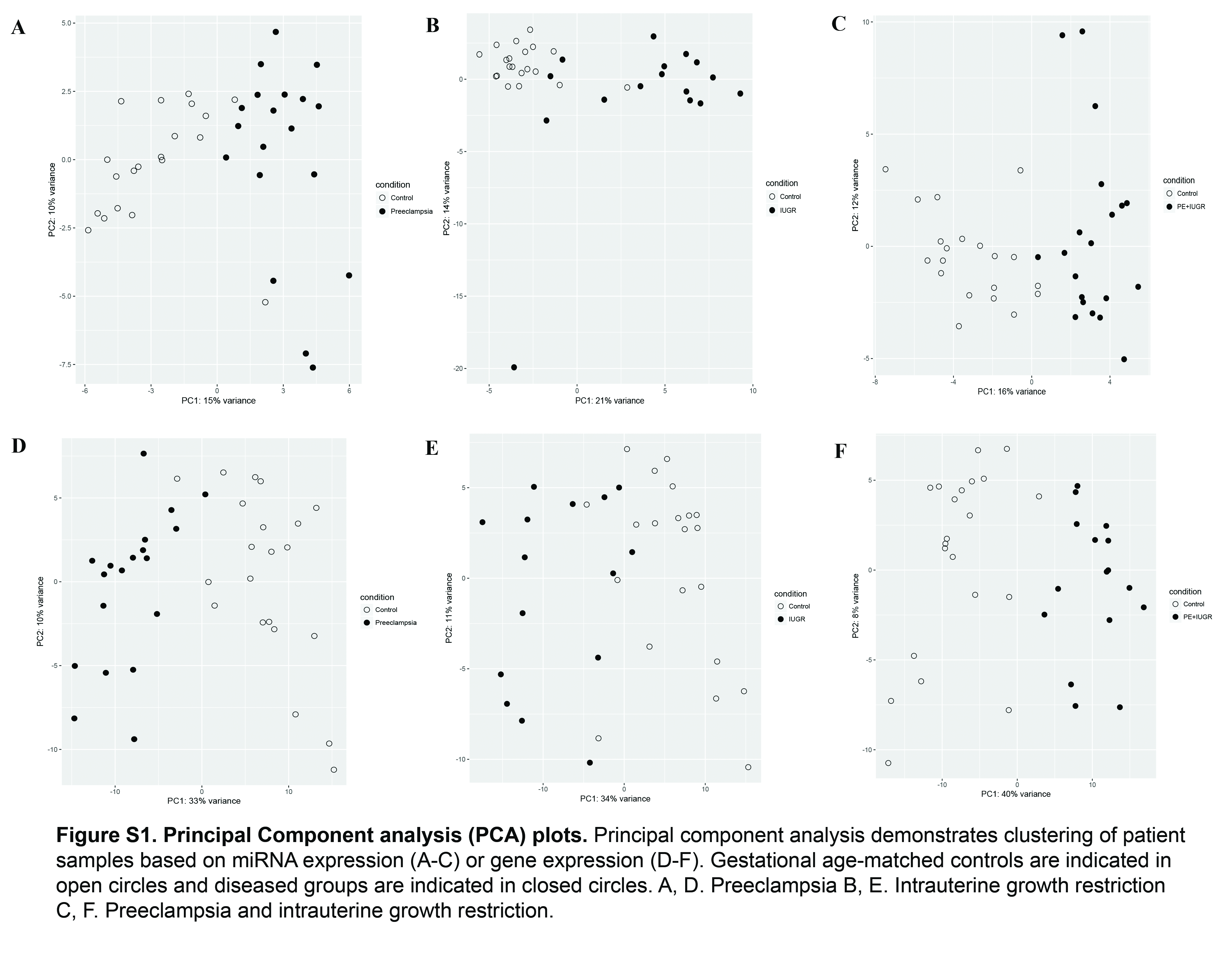

Supplement: Supplementary file 2 — Figure S1. Principal Component analysis (PCA) plots based on miRNA and gene expression datasets. (TIF 17 kb) [file 12920_2019_548_MOESM2_ESM.tif]

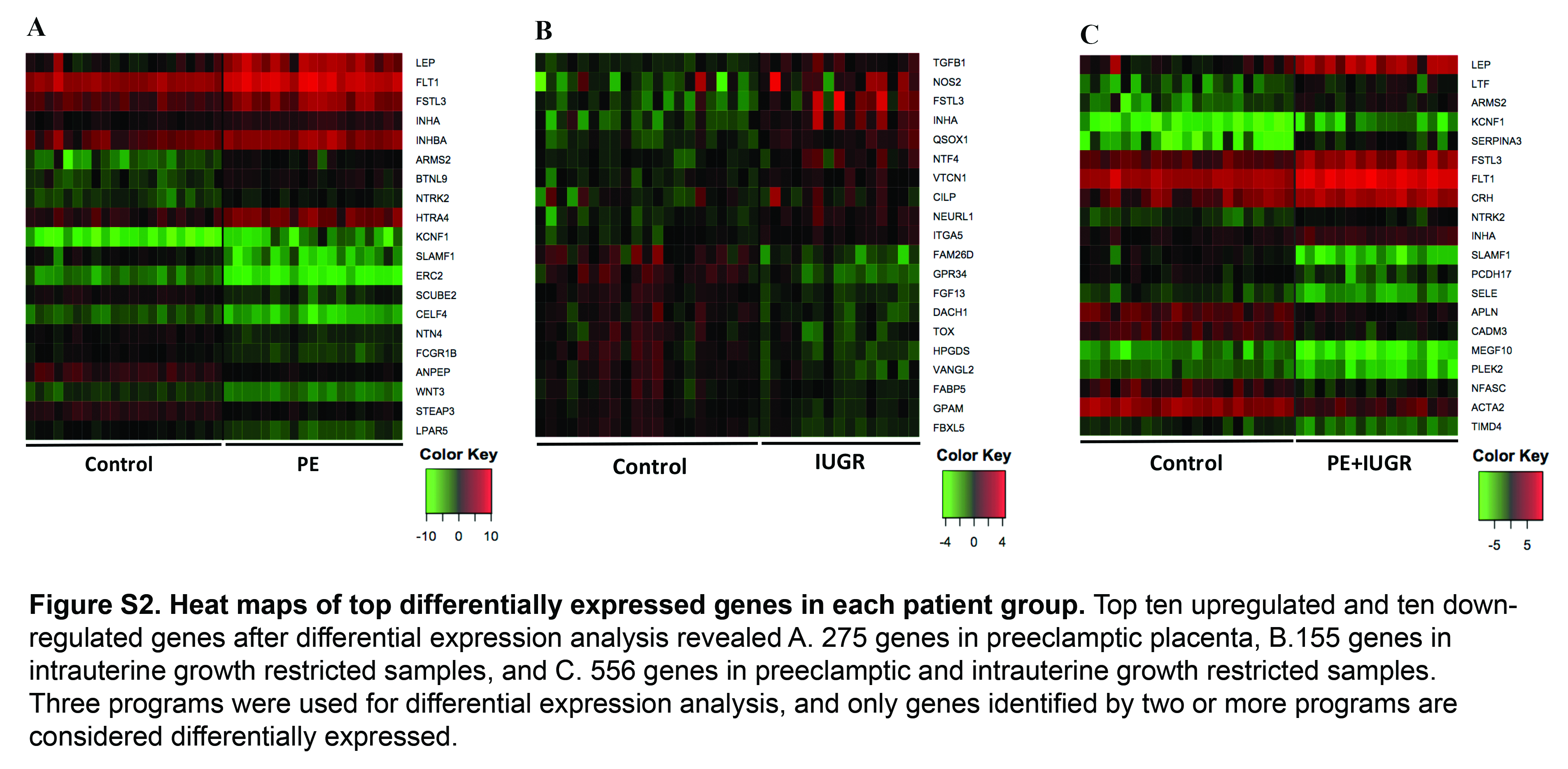

Supplement: Supplementary file 5 — Figure S2. Heat maps of top differentially expressed genes in each patient group. (TIF 12 kb) [file 12920_2019_548_MOESM5_ESM.tif]

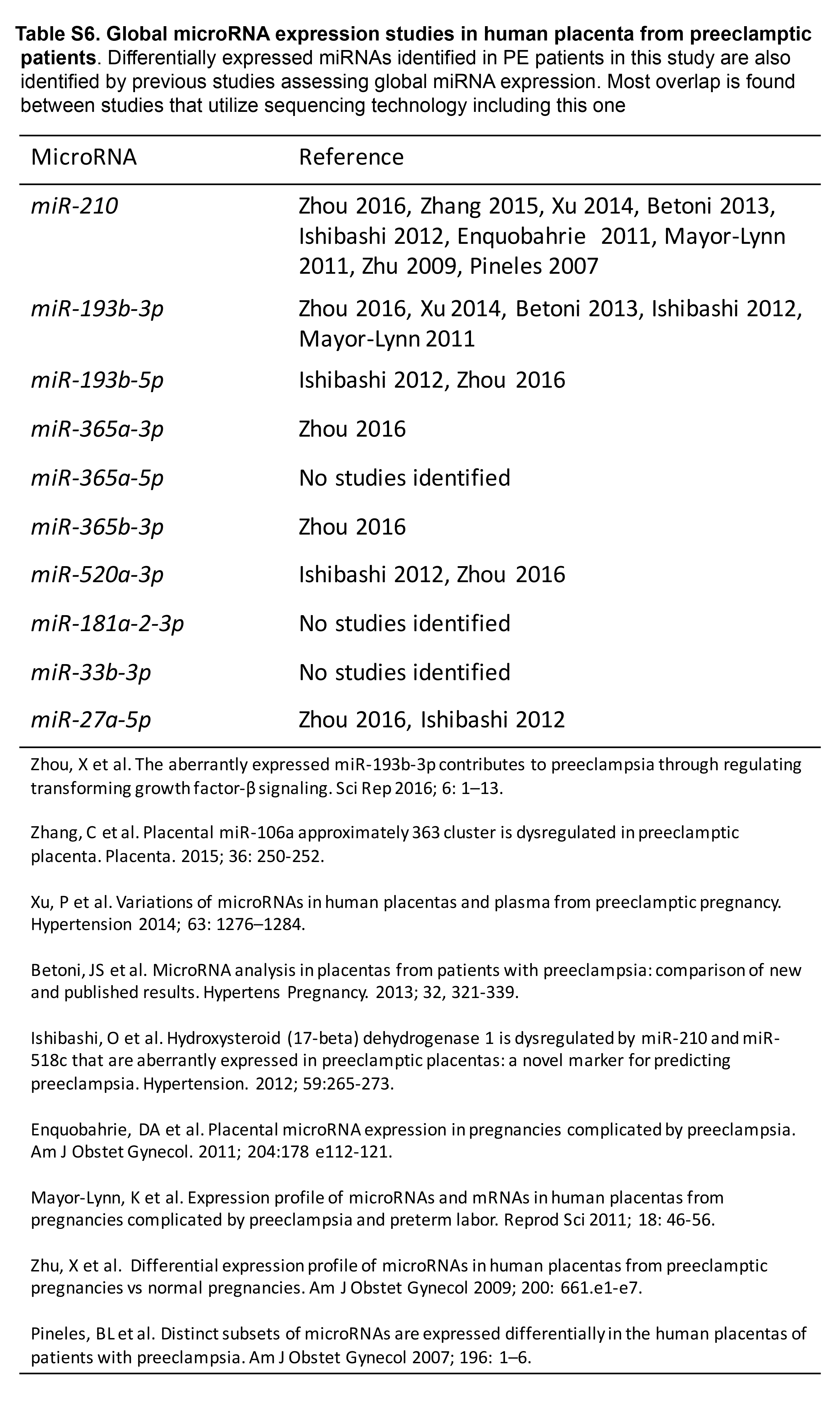

Supplement: Supplementary file 8 — Table S6. List of global microRNA expression studies in human placenta from preeclamptic patients compared to this current study. (TIF 1 kb) [file 12920_2019_548_MOESM8_ESM.tif]
